# The Endophyte *Pantoea alhagi* NX-11 Alleviates Salt Stress Damage to Rice Seedlings by Secreting Exopolysaccharides

**DOI:** 10.3389/fmicb.2019.03112

**Published:** 2020-01-22

**Authors:** Liang Sun, Peng Lei, Qian Wang, Junjie Ma, Yijing Zhan, Kang Jiang, Zongqi Xu, Hong Xu

**Affiliations:** ^1^State Key Laboratory of Materials-Oriented Chemical Engineering, Nanjing Tech University, Nanjing, China; ^2^College of Food Science and Light Industry, Nanjing Tech University, Nanjing, China; ^3^Jiangsu National Synergetic Innovation Center for Advanced Materials, Nanjing Tech University, Nanjing, China; ^4^Nanjing Institute for Comprehensive Utilization of Wild Plants, China Co-op, Nanjing, China; ^5^Nanjing Shineking Biotech Co., Ltd., Nanjing, China

**Keywords:** endophyte, *Pantoea alhagi*, exopolysaccharides, salt stress amelioration, rice

## Abstract

Endophytes have the potential to enhance the ability of plants to resist salt stress, improving crop development and yield. Therefore, in this study, we isolated an endophyte that produced large amounts of exopolysaccharides (EPSs) from the roots of sea rice and examined its effects on the physiological responses of rice (*Oryza sativa* L. ssp. *japonica* “Nipponbare”) seedlings to salt stress using hydroponic experiments. The endophyte was named *Pantoea alhagi* NX-11 based on its morphological characteristics and 16S ribosomal DNA (rDNA) sequence alignment. Rice seedlings that had been inoculated with *P. alhagi* NX-11 exhibited a 30.3% increase in fresh weight, a 28.6% increase in root length, a 51.6% increase in shoot length, and a 26.3% increase in chlorophyll content compared with control seedlings under normal conditions. In addition, inoculated rice seedlings had a 37.5% lower malondialdehyde content, a 133% higher K^+^/Na^+^ ratio, and a 52.8% higher proline content after 7 days under salt stress, as well as up-regulated expression of proline synthase, down-regulated expression of proline dehydrogenase, and enhanced antioxidant enzyme activities. Interestingly, rice seedlings that were inoculated with an EPS-deficient strain named NX-11^eps–^ that was obtained by atmospheric and room temperature plasma (ARTP) mutagenesis were damaged by salt stress and had similar physiological and biochemical indicators to the control group. Therefore, we speculate that the ability of *P. alhagi* NX-11 to enhance the salt tolerance of rice seedlings is related to the EPSs it produces.

## Introduction

In modern agricultural production, soil salinization is a common abiotic stress that reduces crop growth and yield. Approximately 20% of the total arable land worldwide has been eroded by salt damage to some degree, and the area of salinized soil is continuing to increase at a rate of 10% per year, with current estimates that more than half of the world’s arable land will be affected by salinization by 2050 (Shrivastava and Kumar, 2015). Therefore, improvement of the salt tolerance of crops is of great importance. Plant growth-promoting bacteria (PGPBs) attach to the roots of plants or enter plant tissues and affect their physiological state, particularly under abiotic or biotic stress conditions, and so can enhance the tolerance of plants to salt stress and reduce salt-induced damage in plant cells. Therefore, the use of PGPBs is considered an environmentally friendly and cost-effective strategy for promoting the growth of plants under salt stress (Nadeem et al., 2014; Li et al., 2017).

Among the various PGPBs that are available, endophytes have attracted much attention due to their unique ecological status. Endophytes can promote plant growth through their interactions with the host plant and are considered more suitable than soil microorganisms because they can interact with the host more directly and exhibit better responses to environmental change (Rosenblueth and Martínez-Romero, 2006). Many endophyte strains have been shown to have remarkable effects on the ability of plants to resist salt stress (Hamilton et al., 2012). However, the mechanism by which they do so is not yet fully understood because it is not easy to monitor the physiological state of endophytes *in situ* and their metabolites have complex effects on the host.

Over the past 20 years, many studies have investigated endophytic bacteria, particularly those in the rhizosphere. These microorganisms, which include species in the genera *Rhizobium*, *Bacillus*, *Pseudomonas*, *Pantoea, Paenibacillus*, *Burkholderia*, *Achromobacter*, *Azospirillum*, *Microbacterium*, *Methylobacterium*, *Variovorax*, and *Enterobacter*, increase the tolerance of their host plants to salt and other abiotic stresses (Grover et al., 2011). Based on the different characteristics of these endophytes, particularly the secretions they produce, researchers have proposed some widely recognized modes of action that assist the host in tolerating salt. For instance, Egamberdieva (2009) found that some endophytes produce the auxin indoleacetic acid (IAA), which directly stimulates the growth of host plants and enhances salt tolerance, while Stajner et al. (1997) showed that some endophytes can also secrete antioxidants, such as catalase, promote the accumulation of abscisic acid, and degrade reactive oxygen species (ROS) in plants, thereby alleviating the oxidative damage caused by salt stress. Ashraf et al. (2004) further showed that the inoculation of plants with endophytes that secrete exopolysaccharides (EPSs) could reduce Na^+^ absorption under salt stress, decreasing its toxic effects, and further stated that this is related to the polyanionic property of EPSs, which makes them capable of binding to cations. However, the effects of endophytes with growth-promoting and salt resistance effects result from a combination of factors and multiple mechanisms, potentially including novel modes of action that have not yet been discovered. Consequently, the analysis of new modes of action for endophytes that improve the host’s ability to resist stress is the focus of current research (Reinholdhurek and Hurek, 2011).

Here, we report for the first time on the effect of the endophyte *Pantoea alhagi* NX-11 in enhancing salt tolerance in rice (*Oryza sativa* L. ssp. *japonica* “Nipponbare”) seedlings. We also investigated the mechanism by which NX-11 enhances salt stress resistance in rice seedlings by evaluating its effects on the growth of rice seedlings under simulated salt stress and measuring various physiological indexes and the expressions of related genes.

## Materials and Methods

### Characteristics of Strain NX-11

An endophyte named NX-11 was isolated from the rhizosphere soil of sea rice on a beach in Lianyungang, Jiangsu Province, China, using a halophilic medium and following the method of Rajput et al. (2013). The characteristics of strain NX-11 were examined with reference to Bergey’s Manual of Systematic Bacteriology (Kreig et al., 1989). Genomic DNA was extracted using a genomic DNA purification kit and utilized as a template for polymerase chain reaction (PCR) amplification of the 16S ribosomal DNA (rDNA) gene using the general bacterial primers 27F (5′-AGAGTTTGATCMTGGCTCAG-3′) and 1492R (5′-TACGGYTACCTTGTTACGACTT-3′). The PCR was performed in 50 μL of reaction mixture containing Taq mix (25 μL), primer 27F (10 nM, 2 μL), primer 1492R (10 nM, 2 μL), DNA template (25 μL), and distilled deionized water (dd-H2O; 19 μL). The PCR conditions were as follows: initial denaturation at 94°C for 5 min; 30 cycles at 94°C for 30 s, 50°C for 30 s, and 72°C for 90 s; and a final elongation at 72°C for 10 min. The PCR products were purified and sequenced with GenScript (Nanjing, China), and the obtained sequences were analyzed using the nucleotide module of the Basic Local Alignment Search Tool (BLASTN) on the National Center for Biotechnology Information (NCBI) website.

### Culture Conditions for NX-11

Strain NX-11 was incubated in seed medium containing 1% peptone, 0.5% NaCl, and 0.3% beef extract at 37°C with shaking at 200 rpm for approximately 24 h until the logarithmic phase was reached (5 × 10^8^ cells mL^–1^). A 3-mL aliquot was then transferred to a 250-mL flask containing 50 mL of fermentation medium consisting of 4% sucrose, 0.5% peptone, 0.5% NaCl, and 0.3% beef extract and fermentation was carried out on a rotary shaker at 200 rpm and 30°C for 48 h. The culture broth was centrifuged at 4,000 *g* and 4°C for 10 min to separate the cells from the supernatant, and the cells were washed at least three times with double sterile water. The cell suspensions were then used for plant inoculation.

### Plant Inoculation and NaCl Treatment

Rice seeds (*O. sativa* ssp. *japonica* “Nipponbare”) that had been provided by the Jiangsu Academy of Agricultural Sciences (Nanjing, China) were surface sterilized with 2.5% sodium hypochlorite for 20 min and rinsed with sterile water three times. The seeds were then germinated at 22°C in the dark, following which they were transferred to half-strength modified Hoagland solution (Lei et al., 2015). After 1 week of hydroponic growth, the rice seedlings were randomly divided into four groups: CK, which was an uninoculated control group; NaCl, which was an uninoculated group that was treated with a hydroponic solution containing 100 mM NaCl after 1 week; NX-11, which was inoculated with a hydroponic suspension of NX-11 containing 10^8^ cells mL^–1^; and NX-11 + NaCL, which was inoculated with NX-11 and treated with NaCl after 1 week. Following treatment, the plants were cultured for a further week. Leaf and root samples were then harvested each day during the third week of culturing for evaluation. The hydroponic solution was replenished every 2 days during the culture period.

The attachment of NX-11 to the roots of the rice seedlings was observed by scanning electron microscopy (SEM). The samples that had been treated with NaCl were first washed in sterilized water to remove any hydroponic particles. The samples from NaCl treatment groups were then fixed with 2% glutaraldehyde in 0.1 M phosphate-buffered saline (PBS; pH 7.2) at 4°C for 4 h, following which they were washed with PBS three times for 15 min each. The samples were then post-fixed with 1% OsO_4_ in 0.2 M PBS (pH 7.2) at room temperature for 4 h and dehydrated through a graded ethanol series (20, 40, 60, 80, and 100% ethanol) for 15 min. Residual ethanol in the samples was replaced with isoamyl acetate through incubation for 1 h (Dastager et al., 2010), and the samples were sputter coated. The colonization patterns of the endophytes on the surface of the roots were then observed under an S4800 scanning electron microscope (Hitachi, Tokyo, Japan) operating at 10 kV.

### Measurement of Plant Growth and Physiological Indexes

#### The Chlorophyll Contents

The chlorophyll contents of the leaves of the rice plants were determined following the method of Lei et al. (2015) with some modifications. Briefly, leaves [0.5 g fresh weight (FW)] were cut into disks and bleached with 50 mL of 80% (v/v) aqueous acetone solution in the dark at room temperature for 48 h. The suspension was then centrifuged at 10,000 *g* for 5 min and the absorbance of the supernatant was measured at 645 and 663 nm. The chlorophyll content (C_Chl_) was then calculated using the formula:

C(mggFW-1)Chl=8.05A+66320.29A645

#### The Na^+^ and K^+^ Contents

The Na^+^ and K^+^ contents of the shoots of the rice plants were determined following the method of Wangsawang et al. (2018) with some modifications. Briefly, shoots (0.5 g FW) were incubated in 1 M HCl for 1 day and the resulting extract was measured with a flame spectrophotometer (ANA-135; Tokyo Photoelectric, Tokyo, Japan). The ion contents of each sample were then estimated using Na^+^ and K^+^ standard curves.

#### The Malondialdehyde (MDA) Content

The malondialdehyde (MDA) content of the shoots of the rice plants was determined following the method of Del Buono et al. (2011) with some modifications. Briefly, shoots (0.5 g FW) were ground in 5% (w/v) trichloroacetic acid (TCA) and the resulting homogenate was centrifuged at 12,000 *g* for 15 min. The supernatant was then mixed with 5 mL of 0.5% thiobarbituric acid (prepared with 20% TCA), the mixture was incubated in boiling water for 25 min, and the reaction liquid was cooled to room temperature and centrifuged at 7,500 *g* for 5 min. The absorbance of the supernatant was then measured at 450, 532, and 600 nm, and the MDA content (C_MDA_) was calculated using the formula:

C(μmolL)1MDA=6.45(A-532-A)600-0.56A450

Where A_532_ is the maximal absorbance of the MDA–TBA complex, A_600_ is the minimal absorbance of the MDA–TBA complex, and A_450_ is the error led by sucrose. The MDA content was then expressed as nmol g^–1^ FW.

#### The Proline Contents

The proline contents of the shoots of the rice seedlings were determined following the method of Bates et al. (1973) with some modifications. Shoots (0.5 g FW) were cut into pieces and placed in a test tube, and 5 mL of 3% sulfosalicylic acid was then added to the tube. The tube was incubated in boiling water for 10 min, and approximately 2 mL of the supernatant was mixed with 2 mL of acetic acid and 3 mL of 2.5% ninhydrin. Following this, the mixture was incubated again in boiling water for 40 min and then extracted with 4 mL of methylbenzene. The absorbance of the extract was measured at a wavelength of 520 nm and the proline content was calculated based on the linear relationship between the absorbance at 520 nm and the proline content.

### Antioxidant Enzyme Assay

Crude enzyme was extracted from the shoots of the rice seedlings following the method of Abedi and Pakniyat (2018). Briefly, shoots (0.5 g FW) were homogenized in 1.5 mL of pre-chilled Tris–HCl buffer (pH 7.5) containing 5% sucrose and 0.1% mercaptoethanol, and the homogenate was centrifuged at 10,000 *g* and 4°C for 20 min. The resulting supernatant was used as a crude enzyme extract to determine the activities of superoxide dismutase [SOD; Enzyme Commission (EC) 1.15.1.1], chloramphenicol acetyltransferase (CAT; EC 1.11.1.6), and peroxidase (POD; EC 1.11.1.7). In addition, the protein content was determined using Bradford’s method and was considered when calculating the enzyme activities.

Superoxide dismutase activity was determined following the method of Abedi and Pakniyat (2018). The reaction system contained 1.5 mL of 0.05 M phosphate buffer, 0.3 mL of 130 mM methionine solution, 0.3 mL of 750 μM nitroblue tetrazolium (NBT) solution, 0.3 mL of 100 μM ethylenediaminetetraacetic acid (EDTA)-Na2 solution, 0.3 mL of 20 μM lactochrome solution, 0.5 mL of distilled water, and 0.1 mL of crude enzyme extract. The reaction was started and maintained under a light intensity of 4000 lx for 20 min. In addition, a reaction in which the crude enzyme extract was replaced with phosphate buffer and maintained under the same light intensity for 20 min was used as a control and another reaction that contained phosphate buffer but was incubated in the dark for 20 min was used as a blank. At the end of the reaction, the absorbance was measured at 560 nm. One unit of SOD activity was defined as the amount of enzyme that inhibited NBT reduction by 50%, and SOD activity was expressed as units mg^–1^ protein.

Chloramphenicol acetyltransferase activity was determined following the method of Abedi and Pakniyat (2018). The reaction system contained 0.1 mL of 2% H_2_O_2_ and 2 mL of 50 mM phosphate buffer (pH 7.0), and the reaction was started by adding 0.1 mL of crude enzyme extract. At the end of the reaction, the absorbance was measured at 240 nm. CAT activity was defined as the decrease in H_2_O_2_ per min and was expressed as nmol min^–1^ mg^–1^ protein.

Peroxidase activity was determined following the method of Zhou and Leul (1998). The reaction system contained 2.9 mL of 0.05 M phosphate buffer, 0.5 mL of 2% H_2_O_2_, 0.1 mL of 2% guaiacol, and 0.1 mL of crude enzyme extract. At the end of the reaction, the absorbance was measured at 470 nm. POD activity was defined as the amount of guaiacol oxidized per minute and was expressed as nmol min^–1^ mg^–1^ protein.

### Quantitative Reverse Transcription PCR (qRT-PCR)

Mitochondrial RNA (mRNA) was extracted from the rice seedlings with the RNAiso Plus Kit (TaKaRa Bio, Inc., Shiga, Japan) and was reverse transcribed using the PrimeScript RT Master Mix Kit (TaKaRa Bio, Inc.) according to the manufacturer’s instructions. RT-PCR was then conducted on a StepOnePlus^TM^ system (Applied Biosystems, Foster City, CA, United States) using SYBR Premix Ex Taq^TM^ II (TaKaRa Bio, Inc., Shiga, Japan). Each reaction contained 10 μL of 2× SYBR^®^ Premix Ex TaqII, 0.8 μL of 10 mM forward primer, 0.8 (μL of 10 mM reverse primer, 0.4 μL of 50 × ROX reference dye, 2.0 (μL of complementary DNA (cDNA), and 6.0 μL of sterilized distilled water, and the following two-step reaction procedure was undertaken: stage 1, 95°C for 30 s; stage 2, 95°C for 5 s, and 60°C for 30 s for 40 cycles. The relative changes in gene expression were calculated with the 2^–ΔΔ*CT*^ method (Winer et al., 1999), using actin as a reference gene (Xue et al., 2009) and setting the relative transcript level of each gene on day 1 (i.e., the mean value at the time of salt treatment) to 1. The primers used in this study were designed using the Primer Premier 5.0 software (Premier Biosoft Intl., Palo Alto, CA, United States) and were validated by PCR using cDNA as a template before being used for qRT-PCR. The sequences of these primers are shown in Supplementary Table S1.

### Extraction and Fourier Transform Infrared (FTIR) Analysis of the Crude EPSs

The NX-11 culture broth was diluted three-fold with ultra-pure water, mixed with diatomite, and filtered by vacuum filtration. The filtrate was then concentrated to one-fifth of the initial volume and the concentrated supernatant was deproteinated three times using Sevage reagent (isoamyl alcohol:chloroform = 1:4, v/v). Before collection, the crude EPSs were precipitated with ethanol (1:4, v/v) at 4°C overnight and the yield was determined after lyophilization. The organic functional groups in the EPSs were evaluated by blending 2 mg of EPS powder with 200 mg of KBr powder and pressing it into a transparent pellet. FTIR spectroscopy was then performed with a Tensor27 spectrometer (Bruker, Germany) in the frequency range of 4000–400 cm^–1^.

### Acquisition of an EPS-Deficient Strain by Atmospheric and Room Temperature Plasma (ARTP) Mutagenesis

An EPS-deficient strain of NX-11 named NX-11^eps–^ was bred through ARTP mutagenesis according to the method of Cao et al. (2017) with some modifications. Briefly, 15 μL of NX-11 culture (1 × 10^7^ cells mL^–1^) was coated on a stainless-steel minidisc that was then placed in an ARTP breeding machine (Wuxi TMAXTREE Biotechnology Co., Ltd., Wuxi, China). The settings of the ARTP system were as follows: (1) radio-frequency power, 100 W; (2) treatment distance between the sample plate and the plasma torch nozzle exit, 2 mm; (3) helium gas flow rate, 10 standard liters per minute; and (4) treatment time, 30 s. After the ARTP treatment, the NX-11 cells were suspended in 1 mL of lysogeny broth liquid medium, spread on solid fermentation plates, and incubated at 30°C for 48 h. Any colonies that developed with small radii compared with NX-11 were then picked as mutants and transferred into a new fermentation liquid medium, in which they were incubated at 30°C and 200 rpm for a further 2 days. The yield of EPSs was then determined following the method of Zhu et al. (2019) and the strain with the lowest EPS yield was named NX-11^eps–^. A secondary metabolite analysis of NX-11 and NX-11^eps–^ was then performed according to Khaled et al. (2019) with some modifications (Supplementary Methods “UPLC-MS Analysis of NX-11 and NX-11^eps–^”).

### Comparison of the Effects of NX-11^eps–^ and NX-11 on the Salt Tolerance of Plants

Rice seeds were sterilized and rice seedlings were grown under the same conditions as outlined in section “Plant Inoculation and NaCl Treatment” with some modifications. After 1 week of hydroponic growth, the seedlings were randomly divided into the following three groups: NaCl-1, which was an uninoculated control group; NX-11 + NaCl-1, which was inoculated with a hydroponic suspension containing 10^8^ cells mL^–1^ of strain NX-11; and NX-11^eps–^ + NaCl, which was inoculated with a hydroponic suspension containing 10^8^ cells mL^–1^ of the mutant NX-11^eps–^. After 1 week of cultivation, the seedlings in all three groups were treated with a hydroponic solution containing 100 mM NaCl and the attachment of NX-11^eps–^ to the roots of the rice seedlings under the NaCl treatment was observed by SEM. During the third week of culture, samples were collected from each seedling and the root length, shoot length, FW, Na^+^, and K^+^ contents, MDA content, proline content, and SOD, POD, and CAT activities were measured.

### Statistical Analysis

All experiments were performed in triplicate and the results are presented as means ±standard errors (SE). Data were analyzed by analysis of variance, followed by Duncan’s multiple range test using SPSS software v.17.0 (SPSS, Chicago, IL, United States) and a significance level of *P* < 0.05.

## Results

### Identification of *Pantoea alhagi* NX-11

The endophyte NX-11, which was isolated from sea rice growing on a beach in Lianyungang, Jiangsu Province, China, was found to be a gram-negative, rod-shaped bacterium that had a moist, smooth surface and secreted a large amount of a viscous polymer when grown in fermentation medium (Supplementary Figures S1A,B). BLAST analysis revealed that the 16S rDNA gene sequence of NX-11 showed the highest identity (100%) with *P. alhagi* LYTR-11Z (Supplementary Figure S1C). Therefore, based on its morphology, physiochemical characteristics, and 16S rDNA sequence (GenBank accession number: MN736635), strain NX-11 was named *P. alhagi* NX-11. NX-11 has been deposited in the China General Microbiological Culture Collection Center (CGMCC NO: 15525).

### *Pantoea alhagi* NX-11 Enhances the Growth of Rice Seedlings Under Salt Stress

**FIGURE 1:**
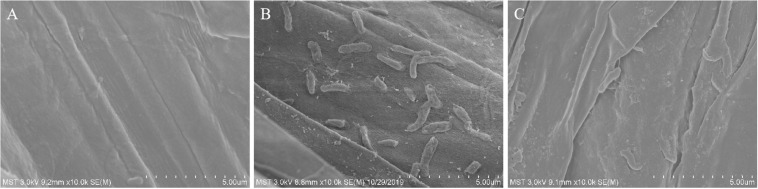
Scanning electron micrographs of the roots of rice (*Oryza sativa* ssp. *japonica* “Nipponbare”) seedlings under salt stress. **(A)** Uninoculated rice seedlings. **(B)** Rice seedlings inoculated with *Pantoea alhagi* NX-11. **(C)** Rice seedlings inoculated with *P. alhagi* NX-11^eps–^.

Scanning electron microscopy observation showed that there were no bacteria on the root surface of plants in the NaCl group (Figure 1A) whereas a number of bacteria were present on the root surface of plants in the NX-11 + NaCl group (Figure 1B).

**FIGURE 2:**
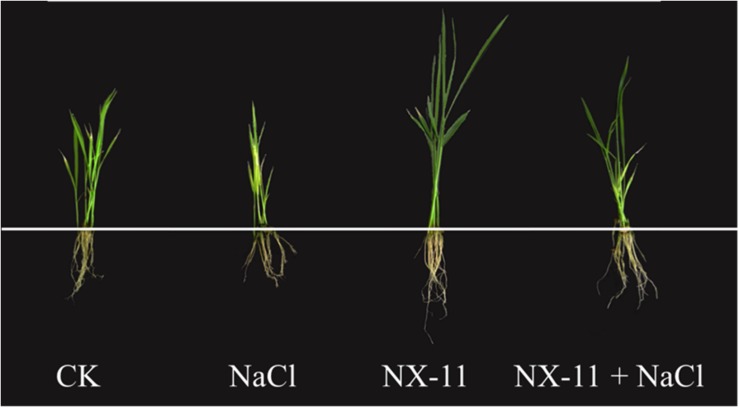
Phenotypes of rice (*Oryza sativa* ssp. *japonica* “Nipponbare”) seedlings inoculated with *Pantoea alhagi* NX-11 (NX-11 and NX-11 + NaCl) and left uninoculated (CK and NaCl) under normal (CK and NX-11) and salt stress (NaCl and NX-11 + NaCl) conditions.

Differences were observed in the phenotypes of plants in the four treatment groups under both normal and salt stress conditions (Figure 2). Rice seedlings in the NX-11 group that had been inoculated with *P. alhagi* NX-11 grew better than uninoculated seedlings in the CK group under normal conditions (0 mM NaCl), exhibiting a 30.3% higher FW, a 28.6% greater root length, a 51.6% greater shoot length, and a 26.3% higher chlorophyll content (Table 1). Following exposure to salt stress for 1 week, uninoculated rice seedlings in the NaCl group exhibited reduced growth and increased leaf chlorosis, whereas inoculated plants in the NX-11 + NaCl group grew normally in the presence of NaCl, exhibiting a 32.6% higher FW, a 30.6% greater root length, a 26.4% greater shoot length, and a 42.5% higher chlorophyll content than seedlings in the NaCl group (Table 1). Together, these findings indicate that *P. alhagi* NX-11 promotes the growth and enhances the salt tolerance of rice seedlings.

**TABLE 1:** The fresh weight, root length, shoot length, and chlorophyll content in rice seedlings inoculated with *Pantoea alhagi* NX-11 and without under normal conditions and salt stress for 7 days*^*a*^*.

Treatment	Fresh weight (g)	Root length (cm)	Shoot length (cm)	Chlorophyll content (mg⋅g^–1^ FW)
CK	0.155 ± 0.004b	11.13 ± 0.04b	20.13 ± 0.04c	0.901 ± 0.007b
NaCl	0.123 ± 0.003c	8.72 ± 0.04c	17.03 ± 0.02d	0.649 ± 0.006c
NX-11	0.202 ± 0.002a	14.31 ± 0.06a	30.51 ± 0.05a	1.138 ± 0.011a
NX-11 + NaCl	0.163 ± 0.003b	11.39 ± 0.05b	21.53 ± 0.04b	0.925 ± 0.006b

**^*a*^*Values are the mean ±SE of 30 replicates. Statistical analysis was performed by Duncan’s test (*p* < 0.05). For the fresh weight, root length, shoot length, and chlorophyll content, the same lowercase letters on each column indicate no significant difference, whereas different lowercase letters on each line indicate a significant difference.*

### *Pantoea alhagi* NX-11 Improves the K^+^/Na^+^ Ratio in the Shoots of Rice Seedlings Under Salt Stress

**FIGURE 3:**
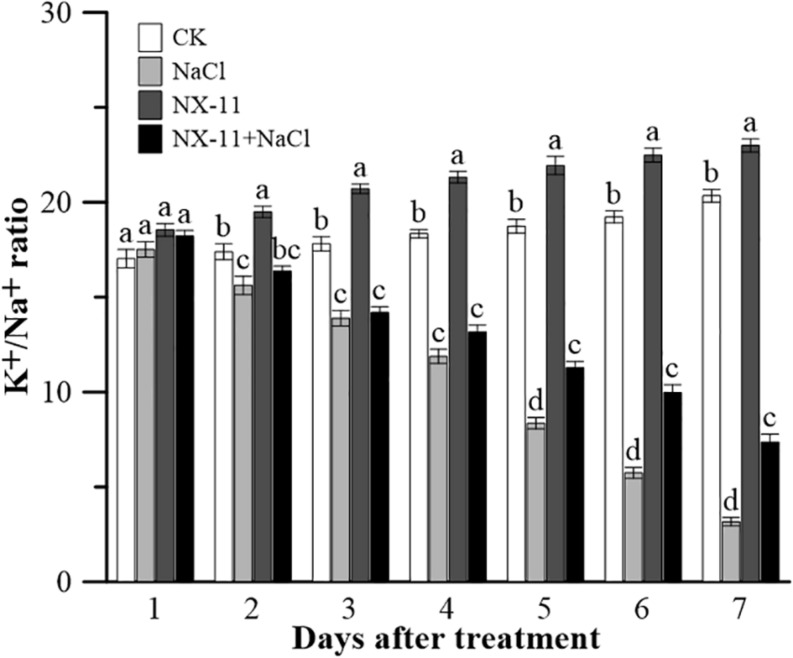
K^+^/Na^+^ ratios in rice (*Oryza sativa* ssp. *japonica* “Nipponbare”) seedlings inoculated with *Pantoea alhagi* NX-11 (NX-11 and NX-11 + NaCl) and left uninoculated (CK and NaCl) under normal (CK and NX-11) and salt stress (NaCl and NX-11 + NaCl) conditions. Values are the means ±SE of three replicates. Different letters indicate significant differences at *P* < 0.05.

Seedlings in the NX-11 group had a higher K^+^/Na^+^ ratio in the shoots than those in the CK group under normal conditions (Figure 3). Salt treatment rapidly decreased the K^+^/Na^+^ ratio, resulting in this being approximately five times lower in seedlings in the NaCl group than in those in the CK group and approximately three times lower in seedlings in the NX-11-NaCl group compared with those in the NX-11 group after 7 days of treatment. However, interestingly, the K^+^/Na^+^ ratio was 133% higher in seedlings in the NX-11+NaCl group than in seedlings in the NaCl group after 7 days of salt treatment.

### *Pantoea alhagi* NX-11 Inhibits the Generation of MDA and Improves the Activities of Antioxidant Enzymes in Rice Seedlings Under Salt Stress

**FIGURE 4:**
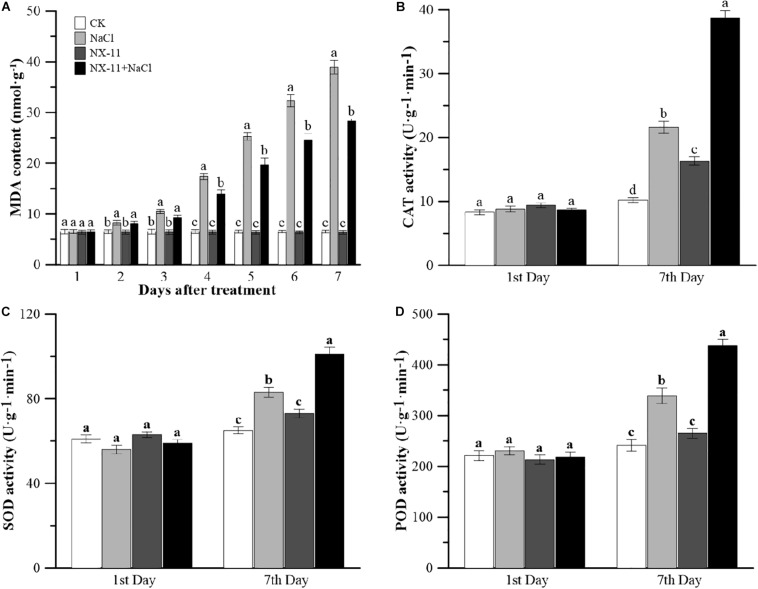
Changes in the malondialdehyde (MDA) content and antioxidant enzyme activities in rice (*Oryza sativa* ssp. *japonica* “Nipponbare”) seedlings inoculated with *Pantoea alhagi* NX-11 (NX-11 and NX-11 + NaCl) and left uninoculated (CK and NaCl) under normal (CK and NX-11) and salt stress (NaCl and NX-11 + NaCl) conditions. **(A)** Changes in the MDA content from the first to seventh days of cultivation under normal and salt stress conditions. **(B)** Comparison of the chloramphenicol acetyltransferase (CAT) activities on the first and seventh days of treatment. **(C)** Comparison of the superoxide dismutase (SOD) activities on the first and seventh days of treatment. **(D)** Comparison of the peroxidase (POD) activities on the first and seventh days of treatment. Values are the means ±SE of three replicates. Different letters indicate significant differences at *P* < 0.05.

Malondialdehyde is the main product of membrane lipid peroxidation, which is closely related to abiotic stress in plants (Miller et al., 2010). Seedlings in the CK and NX-11 groups contained low levels of MDA, with no significant difference between the two groups (Figure 4A). Salt stress had a huge impact on the MDA content, which was almost eight-fold higher in the NaCl group than in the CK group and was also much higher in the NX-11 + NaCl group than in the NX-11 group. However, the MDA content of the seedlings in the NX-11+NaCl group was significantly lower than that of seedlings in the NaCl group throughout the cultivation period and was 37.5% lower on day 7.

The activities of CAT, SOD, and POD showed similar trends in seedlings in the CK and NX-11 groups, with no significant differences between the two groups (Figures 4B–D). The activities of all three of these antioxidant enzymes increased rapidly under salt stress and were consistently higher in plants grown under salt stress (100 mM NaCl) than in plants that were cultivated without salt (0 mM NaCl). Furthermore, the activities of all three enzymes were higher in seedlings in the NX-11+NaCl group than in those in the NaCl group (Figures 4B–D), showing that *P. alhagi* NX-11 can increase the antioxidant capacity of rice seedlings.

### *Pantoea alhagi* NX-11 Increases the Accumulation of Proline and the Expression of Proline Synthetases but Decreases the Expression of Proline Dehydrogenase

**FIGURE 5:**
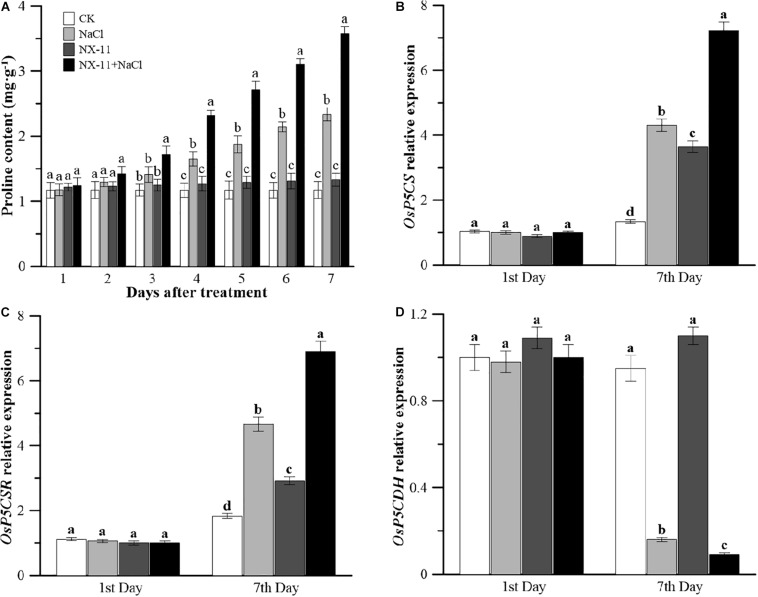
Changes in the proline contents and metabolic gene expression levels in rice (*Oryza sativa* ssp. *japonica* “Nipponbare”) seedlings inoculated with *Pantoea alhagi* NX-11 (NX-11 and NX-11 + NaCl) and left uninoculated (CK and NaCl) under normal (CK and NX-11) and salt stress (NaCl and NX-11 + NaCl) conditions. **(A)** Changes in the proline content on the first and seventh days of treatment. **(B)** Comparison of the *OsP5CS* expression levels on the first and seventh days of treatment. **(C)** Comparison of the *OsP5CR* expression levels on the first and seventh days of treatment. **(D)** Comparison of the *OsP5CDH* expression levels on the first and seventh days of treatment. Values are the means ±SE of three replicates. Different letters indicate significant differences at *P* < 0.05.

Under normal conditions, inoculated seedlings in the NX-11 group had a higher proline content than uninoculated seedlings in the CK group (Figure 5A). Under salt stress, the proline content of the rice seedlings increased sharply, with the proline content of uninoculated plants in the NaCl group being 2.3 times higher than that of seedlings in the CK group after 7 days of salt treatment. This difference was even greater in plants that had been inoculated with NX-11, with seedlings in the NX-11 + NaCl group showing a 3.5-fold higher proline content than those in the NX-11 group and a 52.8% higher proline content compared with uninoculated seedlings in the NaCl group after 7 days of salt treatment.

Under normal conditions, uninoculated seedlings in the NX-11 group had up-regulated expression levels of *OsP5CS* and *OsP5CR*, which are key genes in proline synthesis, and a down-regulated expression level of *OsP5CDH*, which is a key gene in proline metabolism, compared with uninoculated seedlings in the CK group. Under salt stress, there was a significant increase in the expression levels of *OsP5CS* and *OsP5CR* and a significant decrease in the expression level of *OsP5CDH* (Figures 5B–D), and this was enhanced by inoculation with *P. alhagi* NX-11, with seedlings in the NX-11 + NaCl group exhibiting up-regulated transcript levels of *OsP5CS* and *OsP5CR* and down-regulated transcript levels of *OsP5CDH* compared with those in the NaCl group.

### FTIR Analysis of the Crude EPSs

**FIGURE 6:**
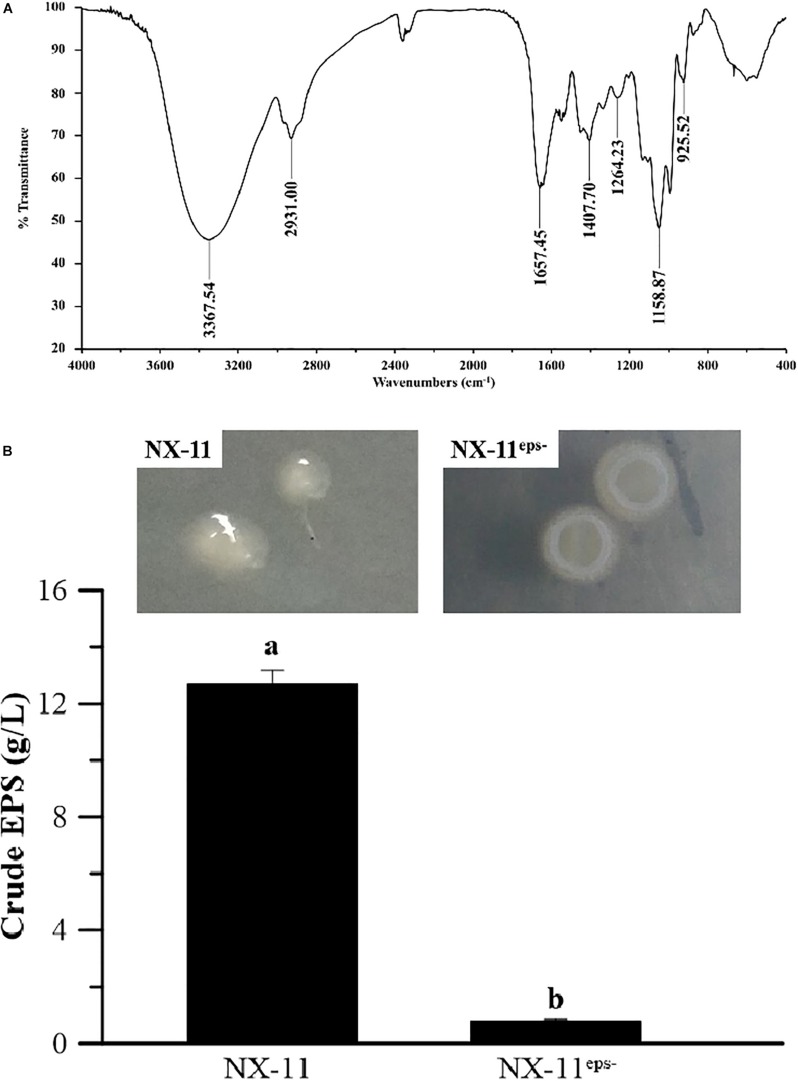
Characteristics of the exopolysaccharide (EPS) secreted by *Pantoea alhagi* NX-11. **(A)** Fourier transform infrared (FTIR) analysis of crude EPS. **(B)** Quantification of crude EPS in NX-11 and NX-11^*eps*–^. Values are the means ±SE of three replicates. Different letters indicate significant differences at *P* < 0.05.

The polymer produced by *P. alhagi* NX-11 was extracted by filtration, deproteinized, and lyophilized, and the functional groups were then analyzed by FTIR (Figure 6A). A broad, highly intense peak was observed at 3367 cm^–1^, which corresponded to the stretching vibration of O-H bonds, while the absorption at 2937 cm^–1^ could be attributed to C-H stretching of CH_2_ or CH_3_ groups. The relatively strong peak at 1657 cm^–1^ indicated the characteristic stretching vibration of the C-O bond (Zhang et al., 2016), and the absorption band at 1407 cm^–1^ was assigned to the bending vibration of the C-H bond. The peak that was observed in the range of 1160–1020 cm^–1^ was related to the presence of C-O-C and C-O-H. Finally, the absorption at 925 cm^–1^ indicated the amount of D-glucopyranosyl (Zhu et al., 2019). These results confirmed that the polymer was an EPS.

### Characteristics of the EPS-Deficient Strain NX-11^eps–^ Obtained by ARTP Mutagenesis

The EPSs produced by the wild type (NX-11) and mutant (NX-11^eps–^) strains were extracted from the culture broth after removing the bacteria, proteins, and water through lyophilization. NX-11^eps–^ produced significantly smaller amounts of EPSs than NX-11 (yield = 0.8 g L^–1^ vs. 12.7 g L^–1^, respectively) (Figure 6B). Therefore, NX-11^eps–^ was selected as the low EPS yield strain and used in subsequent experiments.

Ultra-performance liquid chromatography-mass spectrometry (UPLC-MS) analysis showed that the composition of the secondary metabolites was consistent between NX-11 and NX-11^eps–^ but the ratio of several substances differed between the two strains (Supplementary Figure S2).

### NX-11^eps–^ Is Unable to Enhance the Salt Tolerance of Rice Seedling

**FIGURE 7:**
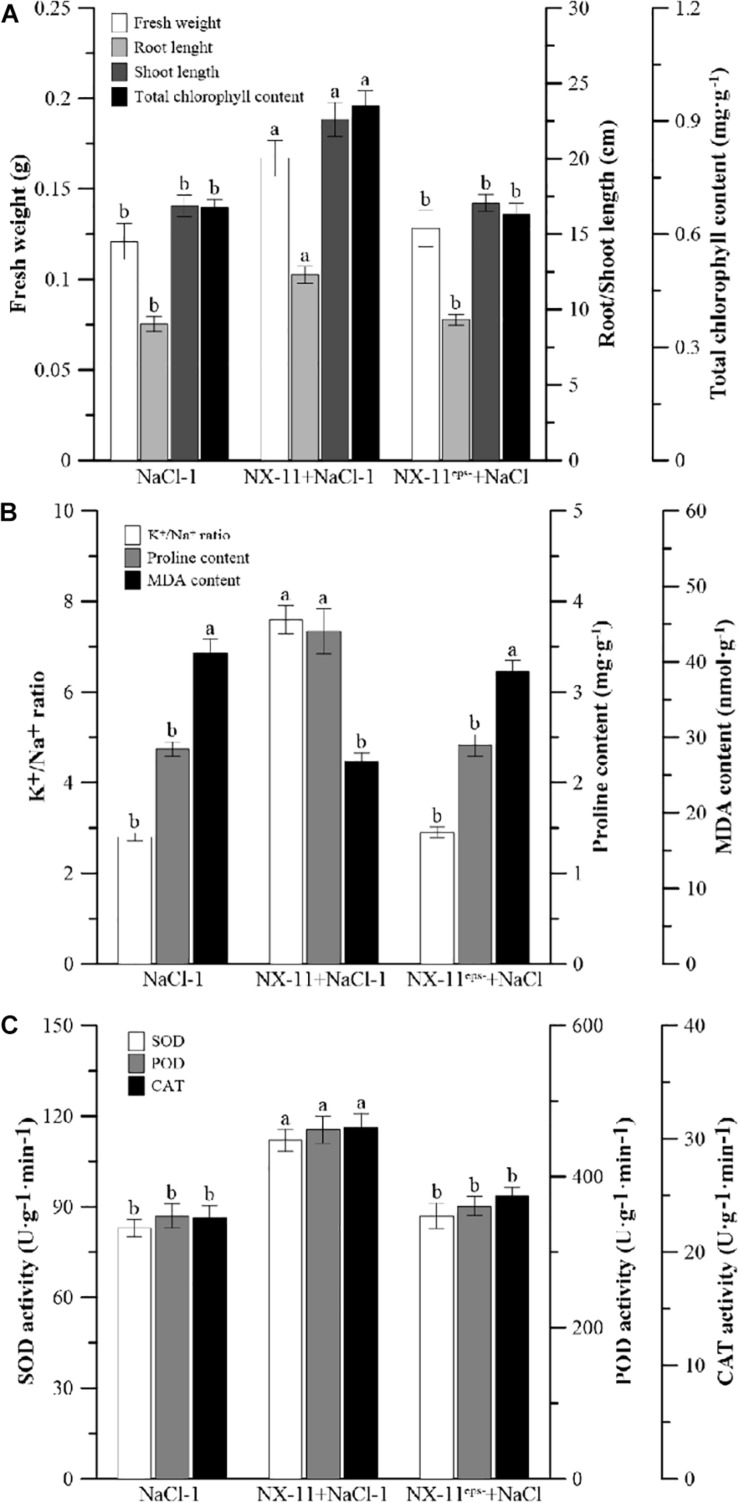
Differences between the inoculated (NX-11 + NaCl-1 and NX-11^eps–^ + NaCl) and uninoculated (CK) rice (*Oryza sativa* ssp. *japonica* “Nipponbare”) seedlings under salt stress. **(A)** Comparisons of the fresh weight, root length, shoot length, and total chlorophyll content on the seventh day of treatment. **(B)** Comparisons of the K^+^/Na^+^ ratio, proline content, and malondialdehyde (MDA) content on the seventh day of treatment. **(C)** Comparisons of the chloramphenicol acetyltransferase (CAT), superoxide dismutase (SOD), and peroxidase (POD) activities on the seventh day of treatment. Values are the means ±SE of three replicates. Different letters indicate significant differences at *P* < 0.05.

Inoculated rice seedlings in the NX-11^eps–^ (+NaCl group exhibited a similar reduction in growth and increased level of leaf chlorosis to uninoculated seedlings in the NaCl-1 group under salt stress, while the seedlings in the NX-11 + NaCl-1 group grew much better than those in the other groups (Figure 7A). It was also found that significantly fewer NX-11^eps–^ bacteria adhered to the rice roots than NX-11 bacteria (Figure 1C). Rice seedlings in the NX-11-NaCl-1 group also had significantly higher K^+^/Na^+^ ratios, proline contents, and SOD, POD, and CAT activities and significantly lower MDA contents than those in the NaCl-1 and NX-11^eps–^ + NaCl groups, which did not significantly differ from each other (Figure 7). Together, these results indicate that the high yield EPS-producing strain NX-11 could enhance the salt tolerance of rice seedlings while the EPS-deficient strain NX-11^eps–^ could not.

## Discussion

Sea rice has a relatively high tolerance to salt, allowing it to maintain a high grain yield under the high salt levels that are encountered on a beach (Yang et al., 2016). However, salt stress impairs the normal physiological, morphological, and biochemical processes of non-halophytes, reducing their growth (Shrivastava and Kumar, 2015). It has been shown that the detrimental effects of salt stress can be effectively alleviated by microbe–plant symbioses, which improve the growth conditions of plants (Porcel et al., 2012). Therefore, increasing numbers of studies are investigating whether endophytes or rhizosphere microorganisms derived from halophytes can enhance the salt resistance of crops and are elucidating their mechanisms of action.

Many studies have shown that the small molecules that are secreted by PGPBs, such as gibberellins, IAA, siderophores, and 1-aminocyclopropane-1-carboxylate (ACC) deaminase, play a role in facilitating plant tolerance to various abiotic stresses (Waqas et al., 2012). In particular, it has been reported that the enhancement of plant stress resistance by PGPBs is related to the secretion of ACC deaminase (Blaha et al., 2006; Barnawal et al., 2012). Salt stress can cause a rapid increase in the ethylene content of plants which, in turn, inhibits their growth (Morgan and Drew, 2010). However, the ACC deaminase that is produced by endophytes can degrade ACC, which is a precursor of ethylene, thereby reducing the ethylene concentration in plants and the adverse effects of excess ethylene on plants and enhancing stress tolerance (Gamalero and Glick, 2012). Based on the results of the BLAST search of the NCBI database and PCR analysis, we predict that *P. alhagi* NX-11 does not secrete ACC deaminase (data not shown), indicating that unlike the bacteria examined in previous studies, NX-11 enhances the salt tolerance of rice via an ACC deaminase-independent mechanism. Interestingly, a large number of the researchers who have proposed that extracellular polymers of PGPBs also play a crucial role in alleviating abiotic stress in plants have focused on polysaccharides and gamma-polyglutamic acid (Livingston et al., 2009; Lei et al., 2017). Here we found that NX-11 secretes large amounts of EPSs (Figure 6B), which benefit the host plants.

Based on the morphological characteristics of NX-11 and phylogenetic analysis of sequences of the 16S region using the neighbor-joining method, we identified this endophyte as *P. alhagi* (Supplementary Figure S1). The genus *Pantoea* is closely related to *Erwinia* (Brady et al., 2008; Supplementary Figure S1C). The first member of *Pantoea* was classified in 1988 and mistaken for *Bacillus agglomerans* (Beijerinck, 1888), and in 1989 Gavini et al. recognized that *Erwinia herbicola*, *Erwinia milletiae*, and *Enterobacter agglomerans* were also synonymous, leading to the transfer of these three groups to the proposed name *Pantoea agglomerans* (Beijerinck, 1888; Beji et al., 1988). Since then, the number of species in the genus *Pantoea* has increased to more than 20 (Walterson and Stavrinides, 2015), some of which have been shown to possess plant growth-promoting capabilities and consequently have been explored for their potential application in agriculture.

To investigate the ability of the PGPB *P. alhagi* NX-11 to promote and enhance salt tolerance in rice seedlings, we conducted hydroponic experiments in which 100 mM NaCl was added to simulate salt stress. These showed that inoculation with *P. alhagi* NX-11 increased the K^+^/Na^+^ ratio and proline content and reduced the MDA content of rice seedlings, counteracting the negative effects of salt stress. Similarly, previous studies have demonstrated that PGPBs can alleviate the damage caused by salt stress in other crops, such as soybean (*Glycine max*) and sunflower (*Helianthus* spp.) (Tewari and Arora, 2014; Lubna et al., 2018). By contrast, the EPS-deficient strain NX-11^eps–^, which was obtained through ARTP mutagenesis, did not exhibit the same ability to alleviate salt stress in rice seedlings and had similar physiological and biochemical indicators to uninoculated seedlings in the control group. UPLC-MS analysis showed that NX-11^eps–^ had a similar composition of secondary metabolites to the wild type NX-11 (Figure 6), although there were slight differences in content (Supplementary Figure S2), demonstrating that the absence of EPS production was the main difference between these two strains. Therefore, we speculate that EPSs play a crucial role in alleviating salt tolerance in crops.

Exopolysaccharides are the main components of the extracellular matrices of microorganisms and so are widely present in PGPBs and affect the growth of crops in many ways. Many studies have shown that PGPBs produce viscous extracellular secretions, such as EPSs, to increase their ability to attach to the rhizosphere of plants. Similarly, we found that *P. alhagi* NX-11 could effectively adhere to the roots of rice seedlings under high salt conditions, whereas NX-11^eps–^ was much less successful in doing so (Figure 1), indicating that the lack of EPSs reduced the ability of these bacteria to adsorb to the roots of plants. EPSs are also capable of maintaining a high moisture content in the rhizosphere due to their high molecular weight, which can also significantly enhance the tolerance of plants under abiotic stress (Silambarasan et al., 2019). In addition, most EPSs are acidic polysaccharides with negative charges and strong adsorption capacities for metal cations, such as Na^+^ and Mn^2+^, allowing them to remove metal ions from their host plant (Mukherjee et al., 2019). This characteristic also explains their amazing ability to mitigate the damage caused by salt stress to crops, because a low Na^+^ concentration or high K^+^/Na^+^ ratio is of great significance to the growth of crops. Supporting this, rice seedlings that were inoculated with *P. alhagi* NX-11 had a significantly higher K^+^/Na^+^ ratio under salt stress than uninoculated seedlings in the NaCl group (Figure 3), whereas rice seedlings that were inoculated with NX-11^eps–^ exhibited a similar K^+^/Na^+^ ratio to seedlings in the control group (Figure 7B). Similarly, Ashraf et al. (2004) showed that endophytic bacteria that were capable of secreting EPSs could alleviate the damage caused by salt stress to wheat (*Triticum aestivum*) seedlings. Interestingly, Tewari and Arora (2014) also found that the inoculation of sunflower seeds with EPS-producing microorganisms increased the germination rate by 50% under salt stress. Thus, the inoculation of plants or seeds with EPS-producing bacteria can alleviate the damage caused by salt stress.

Salt stress causes osmotic stress, which can lead to cell dehydration. Plants usually regulate osmotic stress by actively accumulating small molecules in their cells, such as proline, monosaccharides, and free amino acids (Krishnan et al., 2008). In particular, many studies have investigated the role of proline as an osmotic regulatory molecule, which have shown that its exogenous application can enhance the growth of plants under salt stress (Yoshiba et al., 1997; Ahmed et al., 2010). In the present study, we found that *P. alhagi* NX-11 significantly promoted the accumulation of proline in rice seedlings under salt stress (Figure 5A). Similarly, Zou et al. (2018) found that exogenous polysaccharides increased the proline content in salt-stressed seedlings, increasing their resistance to the salt stress, and Yu et al. (2017) showed that the accumulation of water-soluble polysaccharides in *Dendrobium officinale* enhanced the salt tolerance of the plants by increasing the intracellular proline content.

The protective effect of proline on plants under salt stress is achieved through an increase in the activity of antioxidant enzymes and thereby the elimination of ROS (Ahmed et al., 2010). It has been well documented that salt stress can lead to high levels of ROS production in plant cells, which causes peroxidization of the lipids in the cell membrane and the production of MDA as the final oxidation product, resulting in severe oxygen toxicity in living cells (Kotchoni et al., 2006). Similarly, rice seedlings that were grown under salt stress were seriously damaged by peroxidation, the most obvious feature of which was the significant increase in MDA content that occurred compared with seedlings growing under normal conditions (Figure 4A). Zou et al. (2018) showed that exogenous polysaccharides could scavenge free radicals and protect plants against lipid peroxidation caused by ROS, though unfortunately they were unable to clarify the mechanism by which they do so. However, Mcainsh et al. (1996) found that low concentrations of H_2_O_2_, which is the most stable type of ROS in plants, do not cause damage to plants but rather can act as signal molecules, and Ortmann et al. (2006) showed that EPSs secreted by the endophyte *P. agglomerans* could enhance H_2_O_2_ accumulation and increase peroxidase activity in a suspension of wheat cells. Ortmann et al. (2006) also observed that wheat cells responded strongly to an EPS elicitor, suggesting that the priming mechanism may act on the intracellular defense signaling cascade. For example, it has been shown that a faster or greater activation of transcription factors triggers the expression of defense genes, enhancing the ability of plants to resist abiotic stress (Conrath et al., 2002), and similar phenomena have been reported in cell suspensions from plants such as rice, tobacco (*Nicotiana tabacum*), and parsley (*Petroselinum crispum*) (Ortmann and Moerschbacher, 2006).

In the present study, we found that rice seedlings that had been inoculated with *P. alhagi* NX-11 exhibited significantly higher activities of the antioxidant enzymes SOD, POD, and CAT than uninoculated seedlings on the 7th day after salt stress treatment, even though the antioxidant enzyme activities of seedlings in the NaCl group were much higher than those of seedlings growing under normal conditions (Figures 4B–D). Similar results were obtained when eggplant (*Solanum melongena*) was inoculated with the PGPB *Pseudomonas* sp. DW1 (Fu et al., 2010). The enhancement of these enzyme activities helps plants to inhibit the peroxidation of membrane proteins and lipids and to reduce the MDA content. By contrast, rice seedlings that were inoculated with NX-11^eps–^ had similar proline and MDA contents and SOD, POD, and CAT activities to seedlings in the uninoculated group.

Together, these results indicate that the EPSs that are secreted by *P. alhagi* NX-11 can effectively increase the K^+^/Na^+^ ratio and remove ROS by increasing the proline content, enhancing the activities of SOD, POD, and CAT, and reducing the MDA content, alleviating the damage caused by salt stress to rice seedlings.

## Conclusion

In this study, we isolated the endophytic bacterium *P. alhagi* NX-11, which produces high levels of EPSs, from the roots of sea rice and found that the inoculation of rice seedlings with this bacterium could effectively alleviate the toxicity of salt stress. Under salt stress, rice seedlings that had been inoculated with *P. alhagi* NX-11 had a significantly higher K^+^/Na^+^ ratio, higher proline content, and lower MDA content than uninoculated rice seedlings. However, rice seedlings that were inoculated with the EPS-deficient strain NX-11^eps–^ were damaged by salt stress. Therefore, we speculate that the alleviation of salt stress damage was related to the EPSs secreted by *P. alhagi* NX-11.

## Data Availability Statement

All datasets generated for this study are included in the article/Supplementary Material.

## Author Contributions

HX conducted the experiments. LS planned and wrote the manuscript. PL conducted the *in silico* analysis. QW helped to perform the experiments and wrote the manuscript. KJ helped to perform the ARTP mutagenesis and collaborated with the LS, PL, QW, JM, YZ, ZX, and HX to correct the manuscript. ZX provided the samples and collaborated with the LS, PL, QW, JM, YZ, KJ, and HX to correct the manuscript. JM implemented the qRT-PCR analysis.

## Conflict of Interest

YZ was employed by company Nanjing Shineking Biotech Co., Ltd.

The remaining authors declare that the research was conducted in the absence of any commercial or financial relationships that could be construed as a potential conflict of interest.
