# Supplementary material for: The Endophyte Pantoea alhagi NX-11 Alleviates Salt Stress Damage to Rice Seedlings by Secreting Exopolysaccharides
Source: Front Microbiol. 2020 Jan 22;10:3112. doi: 10.3389/fmicb.2019.03112 (PMC6987256; doi:10.3389/fmicb.2019.03112)
Supplement: Supplementary file 1 [file Data_Sheet_1.docx]

**Supplementary Material**

1. **Supplementary Methods**

**1.1 UPLC-MS Analysis of NX-11 and NX-11^eps-^**

The strains, NX-11 and NX-11^eps-^, were incubated at 37 °C, 200 rpm for 24 h in a 5 mL LB medium, respectively. The supernatant was separated by centrifugation at 4 °C, 5,000 × *g* for 10 min and was used to extract the secondary metabolites by ethyl acetate ([Yuyama et al., 2017](#_ENREF_1)). The secondary metabolites was dried by evaporation at 40 °C in a rotary vacuum evaporator

UPLC-MS analysis was carried out on an Acquity I Class UPLC system (Waters Crop., Milford, MA, USA) coupled with a Mass QTOF mass detector (7890B, Agilent Technologies Inc., California, USA) and an Acquity UPLC BEH C18 column (100 mm × 2.1 mm, 1.7μm). Chromatographic and mass spectrometry conditions were the same as described by Zhao et al. with some modifications ([Zhao et al., 2019](#_ENREF_2)). Briefly, 5 μL ethyl acetate extract was separated on the column with the mobile phases of 0.1% formic acid in acetonitrile (A) and 0.1% formic acid in water (B) at a flow rate of 0.40 mL/min. The mass detection was performed in the ESI(-) mode with a mass range from 100 to 1200 Da. The source parameters were as follows: capillary voltage, 2.5 kv; sample cone, 40 V; source offset, 80 V; source temperature, 100°C; flow rate of cone gas, 50 L/h; temperatures and flow rate of desolvation gas (N_2_), 400 °C and 800 L/h; and collision energy, 2 eV in the low energy function and 10 to 30 eV in the high energy function.

1. **SUPPLEMENTARY TABLES AND FIGURES**
   1. **Figures**


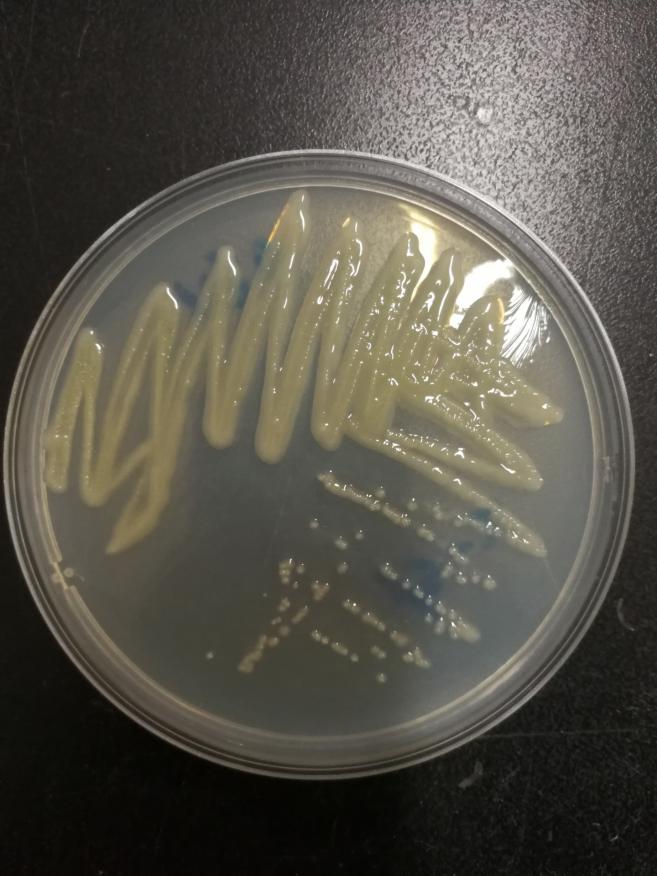

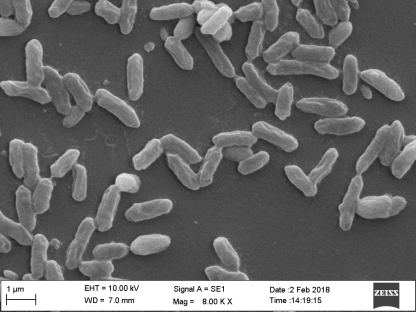

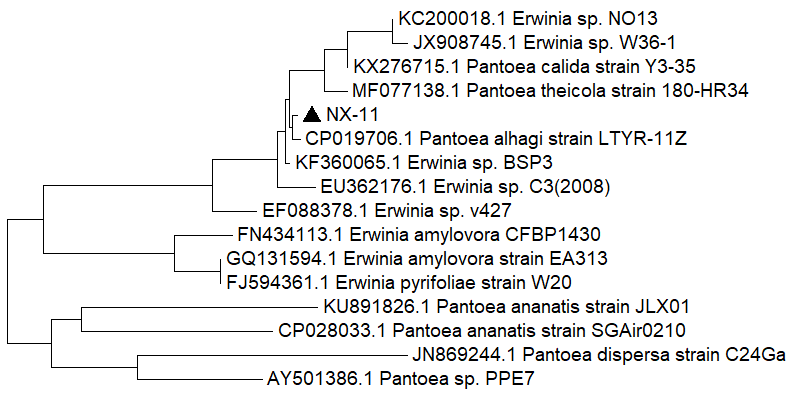


A

B

C

**Figure S1. Morphological characterization and identification of *Pantoea alhagi* NX-11.** (A) Colony morphology of NX-11. (B) Scanning electron micrograph of NX-11 colonies. (C) Phylogenetic analysis of 16S rDNA sequences related to NX-11.

**
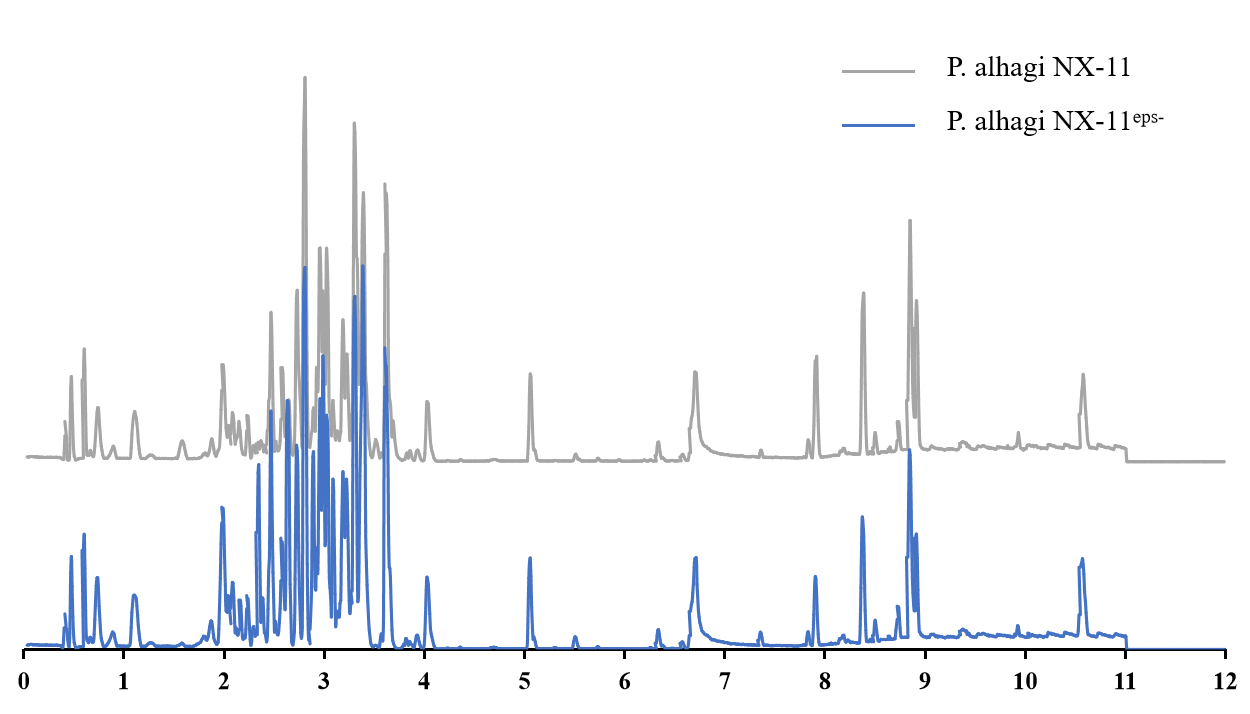
Figure S2. Difference in composition and content of secondary metabolites between *P. alhagi* NX-11 and *P. alhagi* NX-11^eps-^**

- 1. **Table**

**Table S1. Primer Sequences Used for qRT-PCR**

| gene | Primer sequence |
| --- | --- |
| *actin* (LOC4338914) | F: 5’-TGGAAGATTGGCTTTGGGCA-3’ |
|  | R: 5’-CCCGGAACTTTGGGTTCTCA-3’ |
| *OsP5CDH* (LOC9269289) | F: 5’-AAGCATCTCTACGTCCATGCC-3’ |
|  | R: 5’-AGCTCCCATTTGGGGTTCTG-3’ |
| *OsP5CS* (LOC4338979) | F: 5’-TGGAAGATTGGCTTTGGGCA-3’ |
|  | R: 5’-CCCGGAACTTTGGGTTCTCA-3’ |
| *OsP5CR* (LOC4325755) | F: 5’-ACGGAAACGAGAAAAACCAGG-3’ |
|  | R: 5’-CCAAGTGACGAGCAAAACCC-3’ |

**References**

Yuyama, K.T., Chepkirui, C., Wendt, L., Fortkamp, D., Stadler, M., and Abraham, W. (2017). Bioactive Compounds Produced by *Hypoxylon fragiforme* against Staphylococcus aureus Biofilms. *Molecules* 5**,** 80-88. doi: 10.3390/microorganisms5040080

Zhao, Q., Shan, G., Xu, D., Gao, H., Shi, J., Ju, C., et al. (2019). Simultaneous Analysis of Twelve Bile Acids by UPLC-MS and Exploration of the Processing Mechanism of Bile Arisaema by Fermentation. *Journal of Automated Methods & Management in Chemistry* 2019**,** 1-16. doi: 10.1155/2019/2980596
